# Training future dentists: Acquisition of “soft skills” during oral medicine clinical chairside teaching in an undergraduate dental school

**DOI:** 10.1002/jdd.13797

**Published:** 2024-12-08

**Authors:** Neha Thakerar, David Dymock, Konrad Spiteri Staines

**Affiliations:** ^1^ Bristol Dental School Bristol UK; ^2^ Bristol Dental School University of Bristol Bristol UK

**Keywords:** critical thinking, patient communication, soft skills, understanding patient perspective

## 1 PROBLEM

“Soft skills” is an umbrella term grouping together essential skills, such as critical judgment, empathy, and communication. Development of such skills is critical for clinicians due to the significant interpersonal contact with patients.^1^

Soft skills enhance the delivery of other technical skills in providing safe, and effective patient care.^2^, ^3^ Indeed, the UK regulator (General Dental Council) refers to soft skills within the standards of conduct, performance, and ethics that govern dental professionals.^4^

Clinical chairside teaching delivered within oral medicine (OM) sessions provides an ideal environment for students to learn soft skills,^5^ particularly due to the nature of the clinical encounter.

## 2 SOLUTION

The aim of this study was to analyze the effect of soft skill teaching as part of clinical chairside teaching in OM clinics, and whether this value is enhanced by an intervention. This intervention was a small group teaching session, delivered utilizing a standardized PowerPoint™, during OM clinics in Bristol Dental School. The Bachelor of Dental Surgery (BDS) course in Bristol is a 5‐year full time undergraduate course. Apart from communication skills in Year 3, soft skills are not formally taught. Ethical approval was gained, students were given participant information sheets to explain the study; participants signed the designated consent form.

Two groups of dental students were recruited. The first group had 46 fifth‐year students (BDS 5) and the second 53 fourth‐year students (BDS 4). Only BDS 4 received the soft skills intervention teaching at the start of their clinical session. Both groups received OM clinical teaching, which involves students clerking patients in a supervised clinical environment. This facilitates practice and development of soft skills at the chairside.

Immediately after the OM clinic, participants completed an online questionnaire accessed via a QR code. This involved questions under three broad themes, each with a linear scale response, designed to obtain the views of students on the soft skills they gained from each session.

The three “soft skill themes” were: (1) Understanding the patient's perspective; (2) Communication with patients; (3) Critical thinking. Data were collected throughout the year and quantitatively assessed.

## 3 FINDINGS

**FIGURE 1:**
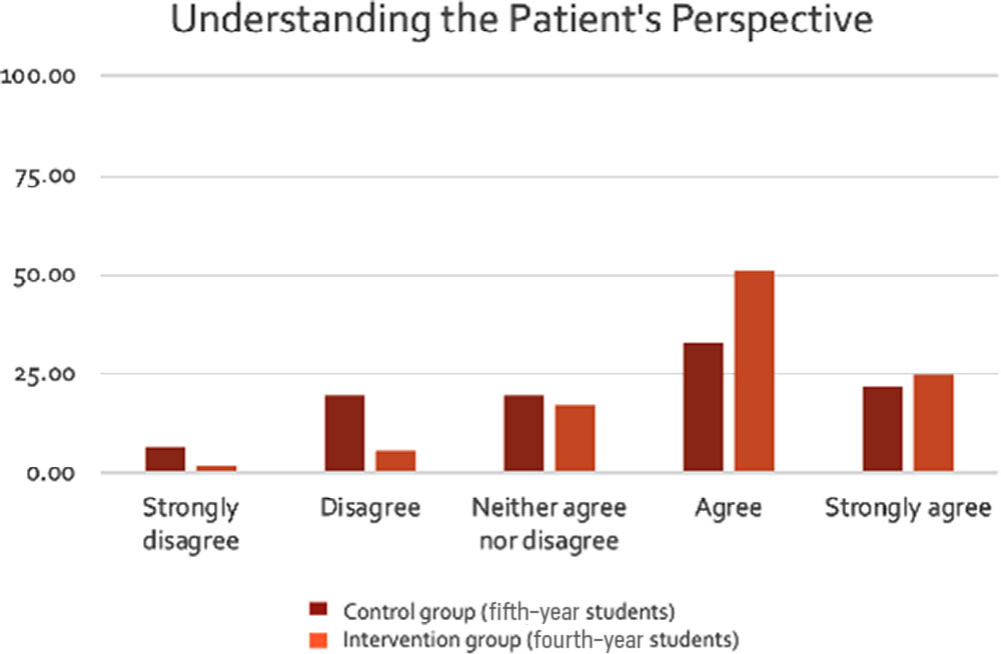
Comparison of responses by students in BDS 4 (intervention) and BDS 5 (control) to the following statements relating to how well they thought they understood the patient's perspective on oral medicine clinics: (1) “I was able to understand the patient's concerns and/or aims of the appointment.” (2) “I was able to understand the primary goal of the patient attending this appointment.” (3) “I was able to gather information and wider knowledge on the patient's personal situation.”

**FIGURE 2:**
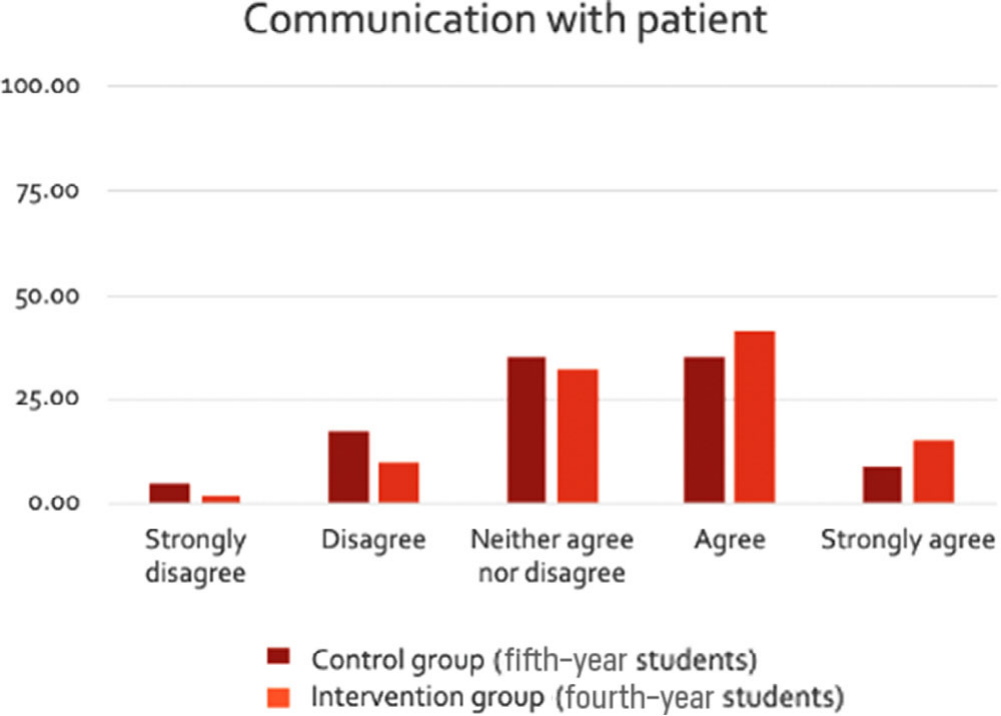
Comparison of responses by students in BDS 4 (intervention) and BDS 5 (control) to the following statements relating to how well they thought they communicated with the patient on oral medicine clinics: (1) “I was able to listen well to the patient and understand their main concerns.” (2) “I was able to empathize appropriately with the patient.” (3) “I was able to manage the patient's expectations appropriately.” (4) “I was able to engage in a shared decision‐making dialog with the patient.”

**FIGURE 3:**
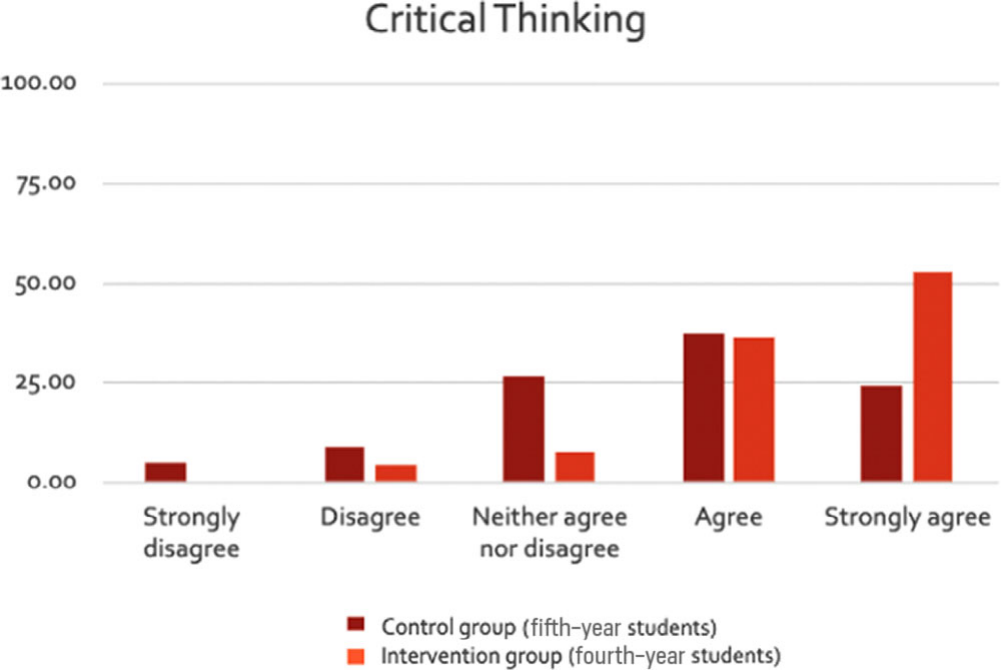
Comparison of responses by students in BDS 4 (intervention) and BDS 5 (control) to the following statements relating to their own critical thinking skills on oral medicine clinics: (1) “I found that presenting the patient's history and examination to supervising clinicians helped me to develop my critical thinking skills.” (2) “I found that answering questions, posed from supervising clinicians, helped me to develop my critical thinking skills.” (3) “I found that observing and comparing different approaches by supervising clinicians to clinical situations, helped me to develop my critical thinking skills.”

Results are presented in Figures 1, 2, 3. The majority of students agreed that their confidence in delivering soft skills had improved; more so for empathy (Figure 1) and critical thinking (Figure 3), than for communication (Figure 2). A higher percentage of students in BDS 4 (intervention) agreed with statements that supported more soft skill development in all three categories compared with BDS 5 (control).

This study provides evidence that OM clinical chairside teaching improves students’ soft skills, which are further enhanced by a focused intervention immediately prior to the OM clinical block. This intervention increased development and awareness in students’ soft skills learning, compared to chairside teaching alone. Developing awareness of “soft skills” as a concept, and identification of these skills as part of clinical care, should be embedded within clinical teaching.

This intervention was designed to meet our own students’ needs, which may limit its generalizability. However, a brief intervention, as described and implemented above, could be embedded more broadly within dental teaching curricula. Limitations include: the absence of true pre–post‐test, variation of teaching within the rotation inherent to a changing patient mix, and comparison of students in differing year groups.
